# Introducing a Psychiatry Clerkship Curriculum Based on Entrustable Professional Activities: an Explorative Pilot Study

**DOI:** 10.1007/s40596-021-01417-y

**Published:** 2021-02-18

**Authors:** Severin Pinilla, Andrea Cantisani, Stefan Klöppel, Werner Strik, Christoph Nissen, Sören Huwendiek

**Affiliations:** 1 University Hospital of Old Age Psychiatry and Psychotherapy, University of Bern, Bern, Switzerland; 2 Institute for Medical Education, University of Bern, Bern, Switzerland; 3 University Hospital of Psychiatry and Psychotherapy, University of Bern, Bern, Switzerland

**Keywords:** Entrustable professional activities, Clerkship, Undergraduate medical education, Workplace-based assessment

## Abstract

**Objective:**

The authors evaluated a reformed psychiatry clerkship curriculum based on entrustable professional activities (EPAs).

**Methods:**

The authors conducted an exploratory pilot study of a reformed clerkship curriculum based on EPAs. A novel workplace-based assessment format including an entrustment-supervision scale and curricular adaptations were introduced. The Kirkpatrick model was used to evaluate outcomes of the reformed clerkship curriculum on three levels (1 = acceptance, 2 = learning, 3 = change of behavior).

**Results:**

The pilot student cohort (*n* = 10) completed a questionnaire, 180 self-assessments (18 per student) on need for supervision, and 63 workplace-based assessments (6.3 per student, in 4 weeks). Level 1: high overall satisfaction with the clerkship (five-point Likert item: average, 4.9; range: 4.0–5.0). Level 2: the overall significant decrease in self-assessed need for supervision before and after the clerkship was two supervision levels (direct to indirect supervision; *p* < 0.05). The most frequently documented admissions included schizophrenic disorders (*n* = 11; 28%), affective disorders (*n* = 10; 25%), substance abuse disorders (*n* = 5; 13%), and anxiety and stress-related disorders (*n* = 5; 13%). Level 3: clinical supervisors used history taking, assessing the mental status, and documentation and presentation for workplace-based assessments. According to supervisors’ ratings, there was a decreasing need for supervision from the first to last week of the clerkship.

**Conclusions:**

Students reacted positively to the reformed clerkship curriculum. The workplace-based assessments with entrustment ratings appeared to support achievement of competency-based learning objectives. Better understanding of how to cover assessment of all core EPAs in the psychiatry clerkship is needed.

Competency-based national frameworks with entrustable professional activities (EPAs) have been introduced in the USA, Canada, and Switzerland for undergraduate medical education [1–3]. EPAs are specific patient care responsibilities that trainees, once they have attained sufficient competence, are “entrusted” to perform unsupervised [4]. The Swiss national learning catalog is based on nine core EPAs that are not specialty specific [1] and are comparable to but still somewhat different than those proposed in the USA by the Association of American Medical Colleges [5]. Currently, in psychiatry, there is only limited evidence available on how to introduce and work with EPAs in clinical training as part of undergraduate medical education [6, 7]. Although two reports are available on assessing EPAs in a psychiatry clerkship curriculum with a rating scale based on expected performance [6, 7], little attention has been paid to using levels of supervision for ad hoc assessment (i.e., entrustment ratings after directly observed clinical activities) of EPAs [8, 9].

The introduction of an EPA-based clinical curriculum typically involves informing students and staff about EPAs as learning goals, for implementation of new teaching activities, and in some cases for reforming workplace-based assessment instruments [10–12]. Observational studies on assessment of EPAs in psychiatry have revealed poor correlation of global performance assessments between student and supervisor ratings per core EPA. Here, students rate their competence higher than supervisors [6], with an expected higher self-assessed level of student performance per EPA later in medical school [7]. Research in other specialties has introduced prospective entrustment-supervision scales for assessment of EPAs, which have not been studied in the context of psychiatry clerkships to date [9, 10]. Although self-assessment of competence is not particularly valuable in terms of validity [13], self-assessment of self-efficacy–related constructs, such as perceived need for supervision or self-entrustment [14], might be more valid in the context of self-regulated learning.

Our objective for this exploratory pilot study was to evaluate educational outcomes after introduction of EPAs, a novel assessment format and associated curricular adaptations to a psychiatry core clerkship curriculum. Therefore, we aimed to explore multiple data sources on the first three Kirkpatrick model levels (satisfaction, learning, and behavioral change) as one of the most widely used evaluation models in medical education [12]. Within this program evaluation, we aimed to pilot self-entrusted supervision levels per core EPA before and after the reformed clerkship rotation [14]. The results from this study can inform future curriculum reforms and might be relevant in particular for clinician educators who are planning to, or are already implementing, EPA-based clinical curricula.

## Methods

We conducted an exploratory pilot study of a competency-based clerkship curriculum reform and assessed the educational curriculum reform outcomes on levels 1–3 of the Kirkpatrick model for program evaluation [12]. The educational concepts that informed our curriculum reform process included entrustment in the workplace [4, 15], and competency-based undergraduate learning and teaching [1, 2].

Medical students at our institution have to complete five mandatory core clerkships during the fourth year of their 6-year medical curriculum (Bachelor and Masters of Medicine). Each year, our psychiatric teaching hospital provides treatment for approximately 3800 inpatients and over 10,000 outpatients. We train about 10 medical students per month, with each teaching ward taking on one or two medical students per month. Students spent 4 weeks on 10 different wards. The interprofessional staff of a ward typically consists of one attending physician and two residents, in addition to nurses, a psychologist, and a social worker, and takes care of 20–22 hospitalized patients. We do not use grades for clerkship rotations but students have to hand in several mandatory formative assessments: ≥4 workplace-based assessments (with at least one on EPA 1 and one on EPA 2); 4 documented patient admissions with active student participation; and 1 scientific paper or clinical case presentation, per student.

On level 1 (i.e., satisfaction), we analyzed the student evaluation of the reformed clerkship curriculum and student in-between and end-of-clerkship feedback. The pilot clerkship rotation with the new curriculum and novel workplace-based assessment format was evaluated by all students with a five-point Likert scale.

On level 2 (i.e., learning), we analyzed the self-entrustment ratings of perceived need for supervision for each EPA before and after the clerkship rotation. To explore the patient-mix exposure as surrogate for learning outcomes [11], we collected students’ documentations of patient encounters, which included patient age, route of admission, and diagnosis.

On level 3 (i.e., behavioral change), we analyzed all of the supervisor-rated workplace-based assessments. These assessment and evaluation data were either collected through paper-based evaluation forms or electronically via ILIAS, throughout February 2019. We used the statistical software R to analyze the supervision scale ratings, with Wilcoxon signed-rank tests.

Students were introduced to the clerkship structure in a reformed clerkship orientation seminar on the first day of their clerkship rotation. In addition to working on the wards and taking part in routine ward and department meetings, students were expected to participate in six didactic clerkship seminars (each seminar lasted one hour, topics covered were: psychopathology, psychiatric interventions, old-age psychiatry, psychosis, stress-related disorders, personality disorders), a scientific paper presentation exercise, and a scientific “journal club.” Furthermore, off-site visits to specialized services, such as addiction-treatment facilities and the child and adolescent psychiatric clinic were part of the curriculum, which also included a patients-as-teachers seminar. Clerkship learning material (e.g., introduction to the electronic medical record system, foundational book chapters, original articles) and knowledge tests for self-regulated learning purposes were provided through a newly integrated online clerkship platform. The sample for this explorative pilot study consisted of 10 medical students with an average age of 22.5 years (nine females, one male). Students were routinely assigned to our teaching hospital by the administrative staff of the medical school. Clerkship directors and supervising clinical staff were introduced to the new workplace-based assessment format with entrustment-supervision ratings in a workshop and via online instruction before implementing the curriculum reform. The new student assessment format included 15 to 20 min direct observation and documentation of the observed EPA, the entrustment rating, and providing narrative feedback.

A clerkship syllabus with nested EPAs for psychiatry (Table 1) was developed by the authors. An open-source learning management system (ILIAS) was used to collect self-entrustment [14] and evaluation data [16].

**Table 1:** Nested psychiatry clerkship EPAs

Psychiatry clerkship nested entrustable professional activity^1^	Self-entrusted need for supervision^2^		
	Pre-clerkship	Post-clerkship	Average change
1) Take a patient’s psychiatric history	2.1	5.5	3.4*
2) Assess mental status	1.8	5.3	3.5*
3) Prioritize a psychiatric differential diagnosis	1.8	3.5	1.7*
4) Order and interpret tests for psychiatric patients	1.4	2.9	1.5*
5) Initiate involuntary treatment	1.1	2.0	0.9*
6) Recognize and treat psychiatric emergencies	1.2	2.5	1.3*
7) Prescribe and develop a management plan for a psychiatric patient	1.3	2.4	1.1*
8) Document and present a clinical encounter with a psychiatric patient	2	5.4	3.4*
9) Identify and report opportunities to improve patient safety in a psychiatric hospital	1.9	2.0	0.1
Overall self-entrustment ratings (*n* = 180)	1.6 (*n* = 90)	3.5 (*n* = 90)	1.9*

^1^Adapated from Principal Relevant Objectives and Framework for Integrated Learning Education in Switzerland (PROFILES)

^2^Self-entrustment of readiness for indirect supervision per entrustable professional activity (six-point Likert items: 1, only observation; 6, indirect supervision)

*p < 0.05, Wilcoxon signed-rank test

**Fig. 1:**
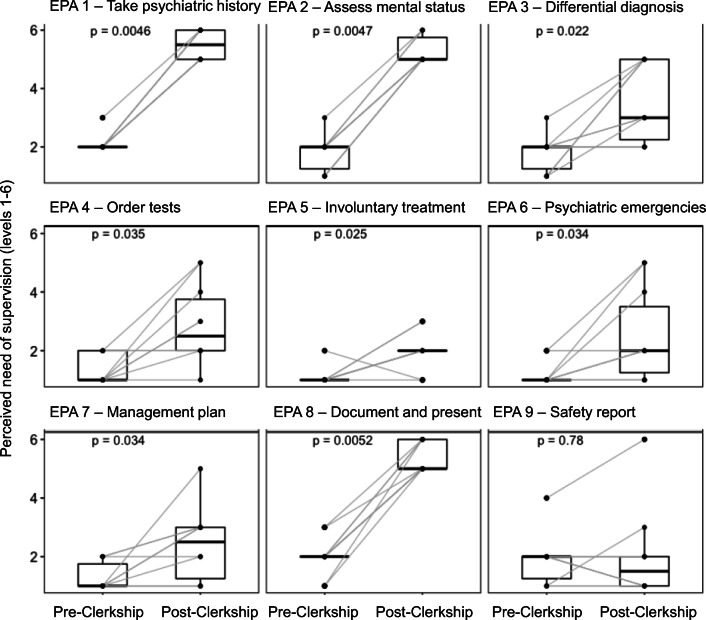
Changes of perceived need for supervision per EPA. Levels of supervision: 1 = “I can only observe this activity,” to 2 = “I can do this only as a co-activity with the supervisor,” 3 = “I can do this activity, if the supervisor is present,” 4 = “I can do this, if the supervisor completely repeats the activity,” 5 = “I can do this, if the supervisor repeats the important parts of the activity,” 6 = “I can do this, if I can ask for help when I need it.” Total of self-assessments: *n* = 180 (90 pre-clerkship, 90 post-clerkship). Statistical analysis: Wilcoxon signed-rank test

Program evaluation data, workplace-based assessments, and self-entrusted need for supervision were collected from all of the medical students in February 2019. We used a published supervision scale for each core EPA [17] for self-entrusted need for supervision (Fig. 1). Each student was asked to rate their need for supervision per core EPA at the beginning and end of the clerkship on a six-point Likert scale (Fig. 1). The Medical Faculty provided an official three-point Likert scale for the supervision on workplace-based assessment forms used throughout the Faculty (1, activity possible with help from supervisor; 2, activity possible with minimal help from supervisor; and 3, activity possible with indirect supervision).

## Results

Level 1 (acceptance): Overall satisfaction indicated agreement with the statement: “I am very satisfied with the overall clerkship experience,” from disagree (1) to agree (5). The overall satisfaction average was 4.9 (range, 4.0–5.0). Students felt well integrated into their ward teams (average, 4.8; range, 4.0–5.0), and they found the online learning platform helpful for their learning experience (average, 4.5; range, 4.0–5.0). They tended to agree with a statement whether the workplace-based assessments were helpful for their learning experience (average, 3.6; range, 2.0–5.0). Seminars and off-site visits were rated from bad (1) to excellent (5), and received an average of 3.5 (range, 2.0–5.0). The positive aspects in the written evaluations by the students mentioned motivation of the attending physicians and residents, and structure and organization of the clerkship curriculum. The students emphasized the importance of structured bedside teaching in the first clerkship week and suggested better instruction of residents with regard to workplace-based assessments and avoidance of redundancy in some of the face-to-face seminars.

Level 2 (learning): The changes in the self-assessed need for supervision differed both across the core EPAs and intra-individually (Fig. 1). The average change of the need for supervision for all nine EPAs was 2.9, which is equivalent to moving from observing an EPA to doing an EPA independently and having a resident check on the activity. However, the self-assessed change in supervision level ranged from 1 to 6. The individual EPA changes (except for EPA 9; Table 1) and the overall change in the self-assessed need for supervision were statistically significant (*p* < 0.05, Wilcoxon signed-rank tests).

Each student participated in and documented four patient admissions (total *n* = 40). Documented admissions included schizophrenic disorders (*n* = 11; 28%), affective disorders (*n* = 10; 25%), substance abuse disorders (*n* = 5; 13%), anxiety and stress-related disorders (*n* = 5; 13%), personality disorders (*n* = 3; 8%), organic illness (*n* = 1; 3%), and eating and impulse control disorders (*n* = 1; 3%), while two admissions did not include any diagnosis (5%).

Level 3 (change of behavior): Clinical supervisors completed 63 workplace-based assessments (average, 6.3 per student). In week #1, 30% (*n* = 19) of the EPA assessments were documented, in week #2, 41% (*n* = 26), in week #3, 17% (*n* = 11), and in week #4, 8% (*n* = 5); two assessments were not dated (3%). The average EPA supervision levels as evaluated by the clinical supervisors per week were: 2.5 (week #1), 2.6 (week #2), 2.5 (week #3), and 3.0 (week #4). Of all of the assessments, 38% (*n* = 24) addressed EPA 1, 43% (*n* = 27) addressed EPA 2, 3% (*n* = 2) addressed EPA 3, 1.5% (*n* = 1) addressed EPA 5, and 14% (*n* = 9) addressed EPA 8 (see Table 1). EPAs 4, 6, 7, and 9 were not used for workplace-based assessments. Students only partly perceived these assessments as relevant for their individual learning progress (average, 3.6; SD, 0.8). With regard to written feedback, 97% of the workplace-based assessment forms contained written feedback narratives.

## Discussion

We conducted an exploratory pilot study to evaluate a reformed competency-based psychiatry clerkship curriculum on levels 1–3 of the Kirkpatrick model [18]. The innovations included EPAs as learning objectives and a novel workplace-based assessment format using an entrustment-supervision scale as well as curricular adaptations (e.g., seminars and digital learning material). In the pilot cohort we found a high overall student satisfaction, a decrease in perceived need for supervision per EPA and a trend towards higher entrustment from the clinical supervisors’ perspective. The changes in self-entrusted need for supervision varied across EPAs and provided insights into which EPAs might be more difficult to master and thus might require additional individual or curricular support. Therefore, self-entrusted need for supervision might be valuable for identifying learners’ needs in workplace-based learning contexts such as clerkships.

A possible explanation for students’ acceptance of competency-based reforms (Kirkpatrick level 1) might be that structuring a clerkship curriculum around EPAs provides a learning scaffold that directly reflects the clinical working environment [19]. To explore learning outcomes of the clerkship students on Kirkpatrick level 2, we used the patient mix, a measure that is typically based on patient volume and patient diversity in terms of diagnoses. In a systematic review, patient mix was shown to correlate positively with self-reported learning outcomes, such as self-confidence, comfort level, perceived quality of the learning experience, perceived effectiveness of the rotation, and instructional quality [11].

While self-assessment of competence is challenging in terms of reliability and objectivity and also not specific in terms of learner needs [13], clinical supervisors can leverage low self-entrustment ratings related to concrete patient care responsibilities (i.e., EPAs) to provide targeted clinical supervision [14]. Changes in perceived need for supervision (also Kirkpatrick level 2) were strongest and most homogenous for those EPAs that were used for workplace-based assessments.

In contrast, EPAs which were not formally observed showed very heterogenous or no changes in self-entrustment. The potential reasons behind these changes might include varying complexity levels of the clinical tasks [20], or inadequate alignment of working context and educational activities and material.

Based on our observations, the deliberate use of formative workplace-based assessments (i.e., assessment for learning instead of assessment of learning) might be a potential driver to achieve learning objectives with regard to EPAs on Kirkpatrick level 3. Since most (71%) of the workplace-based assessments occurred in the first two weeks of a 4-week clerkship our results likely underestimate the students’ performance level at the end of the clerkship. Further research into strategies to effectively support students in mastering core EPAs is needed. Studies and educational reports from psychiatry and other specialties have revealed similar results concerning student satisfaction, improvements in self-confidence in patient care activities and challenges with sufficient workplace-based assessments in the context of EPA-based curricula in undergraduate medical education [10, 14, 15].

The present pilot study is limited by the small sample size, and these preliminary results need to be confirmed. Further research is needed to better understand how and why or why not core EPAs are chosen by supervisors for workplace-based assessments in psychiatry. However, to our knowledge this is one of the first pilot studies that used a systematic evaluation strategy to explore an early EPA-based clerkship curriculum reform.

In conclusion, EPAs as specific patient care responsibilities and the corresponding workplace-based assessments might be used to effectively guide clerkship curriculum design and support students’ attainment of competency-based learning objectives.

## Data Availability

Anonymized quantitative data can be made available from the authors on request.
